# The New Local Hospital Committees

**Published:** 1921-08-06

**Authors:** 


### THE NEW LOCAL HOSPITAL COMMITTEES.

The letters which have been addressed during the past week by the Voluntary Hospitals Commission to the clerks of county councils and county boroughs, requesting their assistance to form the Local Voluntary Hospitals Committees, do not add a great deal of light to that which we have already on the new bodies. But they are taking form, and therefore we shall soon have to be working with them. The general unit will be the county, or the large county borough, and some counties with few hospitals will be formed into single groups. That is the plan for England, but, though the principle is the same, the details will be different in Scotland and Wales. We now pass to the composition of a Local Committee itself. It will consist primarily of certain members, to which not more than five additional members will be nominated by the Commission. Of the first members, as a rule, the county council will nominate two members and each county borough within the county one. The local medical committees in the area will nominate two members, one of whom must be a member of a voluntary hospital's staff and the other a general practitioner. Besides these, the larger general hospitals will nominate one non-medical member, and the smaller and cottage hospitals another. The Commission will nominate not more than five additional members from the residents of the area, one at least of whom must be a woman; and the Committee will choose its own chairman, either from among its members or from outside.

The functions of the Local Committee will bear re-examination, since gradually they are becoming more precisely defined. The first duty of a Local Committee will be, of course, to advise the Commission in regard to the distribution of the grant in its area, and the rest of its duties can almost be described as the actions necessary to discover and to meet the needs of the different hospitals. The Committee will begin by collecting information, though, as we pointed out last week, this is not likely to be accurate, so long as all hospitals do not adopt a uniform financial year and the uniform system of accounts. We note, therefore, with satisfaction that, besides collecting information and furthering co-operation, it will be the function of a Local Committee to "advise as to the adoption of a uniform system of hospital accounts throughout the area." It is humiliating that, at this time of day, with the uniform system long established and strongly commended by the great Funds, such a duty should still be necessary. We trust that Local Committees at any rate will see that the adoption of a uniform system of accounts requires the adoption of the same financial year also. It will be seen that the above clause is very tentative. The Local Committee is not "to advise" the adoption of a uniform system, but to "advise as to" its adoption merely. However, the counsel of a Local Committee which advised *against* the uniform system would make interesting reading. The Local Committee has also to consider the possibility of transferring patients where this can be done with advantage, to prepare where possible schemes of co-operative purchase, and to organise regular contributions from employers and employed where they do not exist at present. It has also to distribute any contributions made by an approved society which is purely local. It will have to co-ordinate appeals.

On paper it would seem at first as if the Local Committees would, if anything, under-represent the hospitals, and until persons chosen by the county councils and the county boroughs are known it would be premature to judge the strength of the Committees. Several of the problems set to the Local Committees are those which the regional committees of the British Hospitals Association are considering at present. With these committees and the Local Committees sufficient energies should be let loose to accomplish on voluntary lines the co-ordination which the voluntary system has made difficult. If the Local Committees are successful we probably have the germ of a better co-ordinated system; but the experience of London seems to show that a central fund exercising the power of the purse is essential if a common standard and common action are to be secured. Something similar to King Edward's Hospital Fund has often been desired for the provinces. Out of the present endeavours, and in a way not yet precisely to be seen, perhaps we may hope eventually to see it. But not too much must be expected, because the grant is so small, and without funds to distribute neither the Commission nor the Local Committees can be much more than advisory bodies.

